# Effects of compound fermentation with *Limosilactobacillus fermentum* HHL-5 and citric acid on wilted king grass silage

**DOI:** 10.3389/fvets.2025.1660833

**Published:** 2025-10-16

**Authors:** Ying Dou, Xindan Xu, Rong Chen, Jinsong Yang, Wei Liu, Haisheng Tan

**Affiliations:** ^1^College of Food Science and Engineering, Hainan University, Haikou, Hainan, China; ^2^College of Materials Science and Engineering, Hainan University, Haikou, Hainan, China

**Keywords:** *Limosilactobacillus fermentum*, wilted king grass, silage, microbial communities, aerobic exposure

## Abstract

To improve the silage quality of wilted king grass, the study investigated the effects of *Limosilactobacillus fermentum* HHL-5 alone and *L. fermentum* complex citric acid fermentation on wilted king grass silage. Four experimental groups were designed as follows: no additive (CK), citric acid addition (CA), *L. fermentum* addition (L), and combined *L. fermentum* and citric acid addition (LCA). The fermentation quality, microbial composition, and aerobic stability of the silage in each group were analyzed. After 30 days of ensiling, LCA had the highest protein content, and L had the lowest ADF content (*p* < 0.05). Lactic acid and acetic acid contents were significantly increased in the LCA group (*p* < 0.05), whereas lactic acid content was increased and acetic acid content was significantly decreased in the L group (*p* < 0.05). Ammonia nitrogen content was significantly decreased in the CA and LCA groups (*p* < 0.05). The L group was not significantly different from the CK group in terms of bacterial diversity and relative abundance, whereas the LCA group showed markedly higher bacterial diversity and was considerably different from the CK group. The relative abundance of *Lactobacillus* in the LCA group was higher than that in the L group, while that of *Enterobacterales* was lower. Compared to the addition of *L. fermentum* alone, complex citric acid silage significantly enhanced the aerobic stability of this feed. In summary, the application of *L. fermentum* combined with citric acid can more effectively improve the quality of wilted king grass silage.

## 1 Introduction

In recent years, the rapid expansion of ruminant farming has significantly increased the demand for roughage. The supply of high-quality roughage is essential for advancing grass-fed animal husbandry, with silage serving as a practical method to ensure year-round feed availability (1). King grass (*Pennisetum purpureum × P. glaucum*, KG) is a hybrid species of the Poaceae family, derived from an intergeneric cross between elephant grass (*P. purpureum*, 2n = 28) and pearl millet (*P. glaucum*, 2n = 14), king grass inherits the beneficial traits of both parent species and is widely recognized as one of the “top high-yield forage grasses”. Its exceptional biomass productivity, wide adaptability, and diverse applications make it an essential economic and ecological resources in tropical and subtropical regions (2). King grass exhibits relatively poor silage quality when directly ensiled. Therefore, the addition of appropriate additives is required for improved preservation. Silage additives are typically classified into two main types: fermentation enhancers and inhibitors. Fermentation enhancers are additives that enhance the fermentation process by lactic acid bacteria. These primarily include fermentable substrates (e.g., sugars and molasses), enzymatic preparations, and specific bacterial inoculants. While fermentation inhibitors increase acidity, lower the pH of silage, and suppress the proliferation of harmful microorganisms. Common inhibitors include inorganic compounds such as aldehydes, salts, and acid–base substances, as well as organic acids such as formic, acetic, citric, and malic acids (3).

In livestock and poultry production, the use of low-dose antibiotics is often unavoidable. Nevertheless, their extensive application in animal feed has led to a range of detrimental consequences, such as antibiotic resistance (4), drug residues in food (5), and environmental pollution (6). Therefore, selecting the appropriate silage additives is crucial for improving feed quality and mitigating these negative effects. In this context, lactic acid bacteria (LAB) are essential for silage fermentation, as they produce organic acids that rapidly lower pH (7), shorten fermentation time, improve feed palatability, and reduce nutrient losses in forage (8). Among the various LAB species, *Limosilactobacillus fermentum* is particularly notable. Difference ordinary lactic acid bacteria, *L. fermentum* strains are highly resistant to acidic and bile conditions (9), making them well-suited for silage fermentation. Research indicates that combinations of propionic acid + *Limosilactobacillus plantarum* + *L. fermentum*, and propionic acid + *L. fermentum* are particularly effective in improving silage fermentation quality. Additionally, the combination of propionic acid + *L. fermentum* has shown superior potential in reducing yeast and mold growth after aerobic exposure, making it an effective approach for improving silage preservation (9). However, research on using *L. fermentum* as an additive is limited, with most studies focusing on *L. plantarum*. One such promising additive is citric acid, which is considered safer and milder than other commonly used acids such as formic or acetic acid (10). Citric acid not only improves feed efficiency, animal health, and productivity (11), but is also cost-effective and can be easily produced through microbial fermentation (12). Studies have demonstrated that adding malic or citric acid, in combination with *L. plantarum*, can improve fermentation quality, limit protein hydrolysis, and enhance the fatty acid composition of alfalfa silage (13). Citric acid also inhibits undesirable microorganisms and works synergistically with LAB to enhance silage quality (13).

Building on this, the present study aimed to assess the physicochemical properties of king grass silage from four treatment groups: without additives (CK), with citric acid (CA), with *L. fermentum* HHL-5 (L), and with the combination of *L. fermentum* and citric acid fermentation (LCA). High-throughput sequencing was employed to analyze the microbial communities in these silage groups, allowing for a deeper understanding of how different conditions affect bacterial populations. Additionally, the aerobic stability and shelf life of the silages were measured, and findings providing a solid theoretical foundation for the research and production of high-quality king grass silage.

## 2 Materials and methods

### 2.1 *Limosilactobacillus fermentum* HHL-5 and citric acid

*L. fermentum* HHL-5: Isolated from the Food Biotechnology Laboratory, School of Food Science and Engineering, Hainan University.

Citric acid: Purchased from China National Pharmaceutical Group Corporation (Sinopharm Group Chemical Reagent Co., Ltd.).

### 2.2 Collection of king grass samples

King grass (Thermal Research No. 4) was collected from the Montenegrin sheep breeding farm at the Institute of Tropical Crop Variety Resources, Chinese Academy of Tropical Agricultural Sciences (CATAS). The collected king grass was chopped into 2–5 cm pieces. After chopping, the grass was transferred to a sterile bags and transported to the laboratory, where it was air-dried within 48 h to achieve a moisture content of less than 65%. Select the period time without precipitation for 7 consecutive days for the sample, and the sampling time was November 2022.

### 2.3 King grass silage preparation and grouping

The experimental design included four groups, as shown in Table 1. The groups were as follows: CK (no additives), CA (citric acid added), L (*L. fermentum* HHL-5 added), and LCA (*L. fermentum* HHL-5 added 0.1% and citric acid added 0.15%). Each group included three replicates, and the sealed silage was stored at room temperature, protected from light. Silage temperature: 30 °C.

**Table 1:** Experimental grouping and inoculation amount.

Group	King grass addition/g	Quantities/bag	Additive quantity
CK	1,000	15	Additive-free
CA	1,000	15	0.15%
L	1,000	15	0.1%
LCA	1,000	15	0.1% + 0.15%

*Limosilactobacillus fermentum* was added at 0.1 (1 × 10^6^ cfu/mL) and citric acid at 0.15%. The percentages are based on the mass of king grass. The silage period lasted for 30 days, with measurements taken at 1, 3, 7, 14, and 30 days. After treatment, the samples were immediately vacuum-packed using a vacuum packing machine.

### 2.4 Analysis of the composition of king grass silage

#### 2.4.1 Nutrient analysis of king grass

Dry matter (DM) content was determined according to the method outlined in GB/T6435-2014 for Determination of Moisture in Feed.

Crude protein content was determined using the Kjeldahl method for king grass silage.

Acid detergent fiber (ADF) content was determined following the method outlined in NY/T 1459-2022 for Determination of Acid Detergent Fiber in Feed.

Neutral detergent fiber (NDF) content was determined according to the method specified in GB/T 20806–2022 for Determination of Neutral Detergent Fiber (NDF) in Feed.

Soluble carbohydrate (WSC) content was determined using the anthrone colorimetric method (14).

#### 2.4.2 Measurement of silage fermentation quality

Determination of silage pH: At each designated sampling time, silage bags from each group were opened, and the upper layer of the sample was mixed. A 10 g portion of the mixed silage was transferred to a 150 mL sterile conical flask, followed by the addition of 90 mL of sterile water. The mixture was stirred until the sample was fully submerged, then sealed. After shaking for 24 h, the mixture was filtered to separate the solid residue, obtaining the leachate. A portion of the leachate was used to measure the pH with a pH meter, while the remainder was stored in a sterile centrifuge tube.

Determination of organic acid content: Centrifuge the prepared silage sample supernatant at 10,000 rpm for 10 min using a high-speed centrifuge to obtain the supernatant. Withdraw the extract using a disposable syringe and filter it through a 0.22 μm cellulose membrane (organic type) to remove impurities. Carefully withdraw the filtered sample using a 10 μL injector, ensuring that no air bubbles are present in the injector. Analyze the sample using high-performance liquid chromatography (HPLC) (15).

Ammonia nitrogen content was determined using the phenol-hypochlorite method (14).

### 2.5 Sequencing the bacterial composition of king grass silage

#### 2.5.1 Extraction of microbial genomic DNA of king grass

For each group of king grass silage, a 25 g of samples was weighed, mixed with 225 mL of sterile water, and incubated in a shaker for 2 h. The silage residue was filtered, and the filtrate was centrifuged for 15 min to collect the precipitate. The microbial genomic DNA was then extracted from the king grass silage samples using the CTBA method.

#### 2.5.2 PCR amplification and sequencing

The bacterial DNA extracted from king grass silage was amplified by PCR. The sequence information of the PCR primers is provided in Table 2, and the PCR reaction system is detailed in Table 3. The amplified products were then sent to Guangdong Mega Gene Technology Co., Ltd. for sequencing.

**Table 2:** Information of primers.

Target gene	Primer sequence (5′-3′)	Product size
16S rRNA V3 ~ V4	515F: GTGCCAGCMGCCGCGGTAA	450
	806R: GGACTACHVGGGTWTCTAAT	

**Table 3:** PCR reaction system.

Reaction system	Addition	Amplification condition	
2 × Premix Taq	25 μL	94 °C 5 min	
Primer-F (10 μM)	1 μL	94 °C 30 s 52 °C 30 s 72 °C 30 s 72 °C 5 min	30 cycles
Primer-R (10 μM)	1 μL		
DNA	50 ng		
Nuclease-free wate	Add to 50 μL		

#### 2.5.3 Bioinformatics and data analysis

Based on the effective data and OTU annotation table provided by the sequencing company, R software was used to analyze the community composition at the kingdom, phylum, class, order, family, genus, and species levels. Alpha diversity analysis was performed using the OTU relative abundance table and the alpha_div command in usearch (V10). A custom Python script (Python v2.7) was used to plot the dilution curve and Rank Abundance curve. PCA analysis was performed using the prcomp function in R, and PCoA plots were generated using the vegan package in R. Species composition and relative abundance information of the research samples were extracted using GraPhlAn software. The sequencing data from this study have been submitted to the National Center for Biotechnology Information Sequence Read Archive database under Bioproject accession number of PRJNA1333438.

### 2.6 Determination of the aerobic stability of king grass silage

After 30 days of silage fermentation, the sample bags were opened, mixed, and placed at room temperature. The pH was measured at 72, 144, and 216 h post-opening to assess aerobic stability based on pH changes.

### 2.7 Data processing and analysis

Experimental data were processed in Excel 2020. Statistical analysis of variance was performed using SPSS 26. Graphs were poltted using Origin 2017. The bacterial and fungal communities sequencing data were generated by the Illumina HiSeq sequencing platform.

## 3 Results

### 3.1 Nutrient analysis of king grass silage

Table 4 presented the nutrient composition of king grass silage for different treatment groups. No significant difference in dry matter (DM) content was observed among the four treatment groups after 30 days of silage compared to pre-silage (*p* > 0.05) (Table 4). The DM content in the LCA group was significantly higher than in the L and CA groups (*p* < 0.05). After 30 days of ensiling, the crude protein content decreased significantly in the CK group compared to pre-silage levels (*p* < 0.05), whereas no significant change in crude protein content was observed in the L, CA, and LCA groups (*p* > 0.05). The crude protein content in the LCA group was significantly higher than that in the CK group (*p* < 0.05). However, no significant difference was observed between the LCA, L, and CA groups (*p* > 0.05). Additionally, there was no significant difference between the crude protein contents of the L and CA groups (*p* > 0.05). This result suggested that the addition of citric acid or *L. fermentum* alone did not reduce crude protein loss during silage. However, combining both additives effectively reduced crude protein loss. After 30 days of ensiling, the contents of NDF and ADF were significantly higher in all four treatment groups compared to pre-silage levels (*p* < 0.05). No significant difference in NDF content was observed among the four treatment groups (*p* > 0.05). However, ADF content was significantly lower in the L, CA, and LCA groups, with the L group showing the lowest ADF content (*p* < 0.05). After 30 days of ensiling, the WSC content was significantly lower in all four treatment groups compared to pre-silage levels (*p* < 0.05). The WSC content ranked from highest to lowest as follows: CK > L > CA > LCA.

**Table 4:** Nutrient composition of king grass silage.

Group	DM (%FM)	CP (%DM)	NDF (%DM)	ADF (%DM)	WSC (%DM)
Pre-storage	37.24 ± 0.55^ab^	11.42 ± 0.73^a^	65.69 ± 1.27^a^	35.03 ± 0.74^d^	6.35 ± 0.26^a^
CK	37.51 ± 0.46^ab^	9.24 ± 0.41^b^	68.74 ± 1.11^a^	44.56 ± 1.22^a^	4.66 ± 0.09^b^
L	35.93 ± 0.73^b^	10.11 ± 0.31^ab^	67.66 ± 1.09^a^	38.99 ± 0.89^c^	3.92 ± 0.07^c^
CA	36.82 ± 0.71^b^	10.04 ± 0.47^ab^	67.48 ± 1.30^a^	40.11 ± 0.86^bc^	2.24 ± 0.05^d^
LCA	38.17 ± 0.53^a^	10.61 ± 0.64^a^	67.17 ± 1.41^a^	41.48 ± 0.48^b^	1.86 ± 0.10^e^

Different lowercase letters indicate significant differences between data in the same column (*P* < 0.05).

### 3.2 Quality and quality analysis of king grass silage

The dynamics of changes in fermentation quality of king grass silage in different treatment groups was shown in Table 5. With the prolongation of silage time, the pH in the four treatment groups of CK, L, CA and LCA showed a decreasing trend. On the 1st day of silage, the pH in the three treatment groups L, CA and LCA was significantly lower than that in the CK group (*p* < 0.05). After 30 days of ensiling, the pH in the CK group was significantly different from that in the three groups of L, CA, and LCA (*p* < 0.05), and the final pH of the four silages of CK, L, CA, and LCA was 4.30, 4.15, 4.06, and 3.97, respectively. Suggesting that the composite additives were better able to reduce the pH of the silage.

**Table 5:** Fermentation quality of king grass ensiled.

Group/project	Clusters	Silage time/d				
		1	3	7	14	30
pH	CK	4.89 ± 0.04^aA^	4.77 ± 0.01^bA^	4.66 ± 0.02^cA^	4.42 ± 0.04^dA^	4.30 ± 0.03^eA^
	L	4.71 ± 0.07^aB^	4.51 ± 0.02^bB^	4.32 ± 0.06^cB^	4.21 ± 0.03^dB^	4.15 ± 0.04^eB^
	CA	4.58 ± 0.03^aC^	4.34 ± 0.05^bC^	4.22 ± 0.02^cC^	4.11 ± 0.03^dB^	4.06 ± 0.04^dC^
	LCA	4.56 ± 0.02^aC^	4.30 ± 0.05^bC^	4.16 ± 0.03^cC^	4.05 ± 0.03^dC^	3.97 ± 0.03^eD^
LA (g/kg DM)	CK	3.08 ± 0.39^aB^	4.56 ± 0.48^bBC^	5.42 ± 0.34^cC^	6.94 ± 0.23^dD^	7.96 ± 0.25^eD^
	L	4.36 ± 0.44^eA^	6.87 ± 0.39^dA^	7.68 ± 0.37^cB^	8.63 ± 0.39^bC^	9.38 ± 0.20^aC^
	CA	2.92 ± 0.46^eB^	3.69 ± 0.34^dC^	5.23 ± 0.40^cC^	18.30 ± 0.39^bB^	30.37 ± 1.12^aB^
	LCA	3.07 ± 0.51^eB^	5.55 ± 0.99^dB^	9.56 ± 0.43^cA^	22.13 ± 1.11^bA^	32.33 ± 1.62^aA^
AA (g/kg DM)	CK	2.09 ± 0.06^dA^	2.98 ± 0.35^cA^	3.34 ± 0.53^bB^	4.52 ± 0.25^aC^	5.62 ± 0.34^aC^
	L	1.57 ± 0.20^eB^	2.25 ± 0.15^dB^	3.29 ± 0.62^cB^	4.11 ± 0.15^bC^	4.69 ± 0.42^aD^
	CA	1.54 ± 0.19^dB^	1.86 ± 0.24^dBC^	4.48 ± 0.38^cA^	5.80 ± 0.24^bB^	14.90 ± 0.48^aB^
	LCA	NA	1.44 ± 0.10^dC^	3.94 ± 0.35^cAB^	10.74 ± 1.01^bA^	16.52 ± 0.43^aA^
Ammonia nitrogen (g/kg DM)	CK	0.104 ± 0.003^bA^	0.113 ± 0.002^aA^	0.087 ± 0.003^cB^	0.088 ± 0.002^cB^	0.085 ± 0.002^cA^
	L	0.094 ± 0.002^cB^	0.112 ± 0.004^aA^	0.102 ± 0.004^bA^	0.105 ± 0.003^bA^	0.084 ± 0.004^dA^
	CA	0.072 ± 0.003^bC^	0.081 ± 0.006^aB^	0.071 ± 0.004^bC^	0.061 ± 0.003^cD^	0.059 ± 0.003^cC^
	LCA	0.075 ± 0.003^aC^	0.063 ± 0.003^cC^	0.072 ± 0.004^abC^	0.066 ± 0.003^bcC^	0.065 ± 0.004^bcB^

Different lowercase letters indicate significant differences between peer data (*P* < 0.05), and different uppercase letters indicate significant differences between data in the same column (*P* < 0.05).

During the 30 days of fermentation, the contents of lactic acid and acetic acid both showed an increasing trend (*p* < 0.05) and reached the maximum value on the 30th day, propionic acid and butyric acid were not detected in the silage. At the end of ensiling, the contents of the lactic and acetic acid differed significantly (*p* < 0.05) among the four treatment groups (CK, L, CA, LCA). The LCA group exhibited the highest concentrations, with 32.33 g/kg of lactic acid and 16.52 g/kg of acetic acid. The analysis of individual effects revealed that both *L. fermentum* and citric acid alone significantly increased lactic acid content compared to CK (*p* < 0.05). Notably, the LCA group exhibited a synergistic effect, yielding significantly higher lactic acid and acetic acid contents than either additive alone (*p* < 0.05), which conclusively demonstrates the superior efficacy of the composite additive in enhancing fermentation quality. Additionally, the ammonia nitrogen content in king grass silage showed irregular changes during fermentation. Overall, the ammonia nitrogen content in groups CA and LCA was significantly lower than that in groups CK and L (*p* < 0.05), while no significant difference was observed between groups CK and L (*p* > 0.05).

### 3.3 High-throughput sequencing of king grass silage

#### 3.3.1 Sequencing data analysis

**Figure 1:**
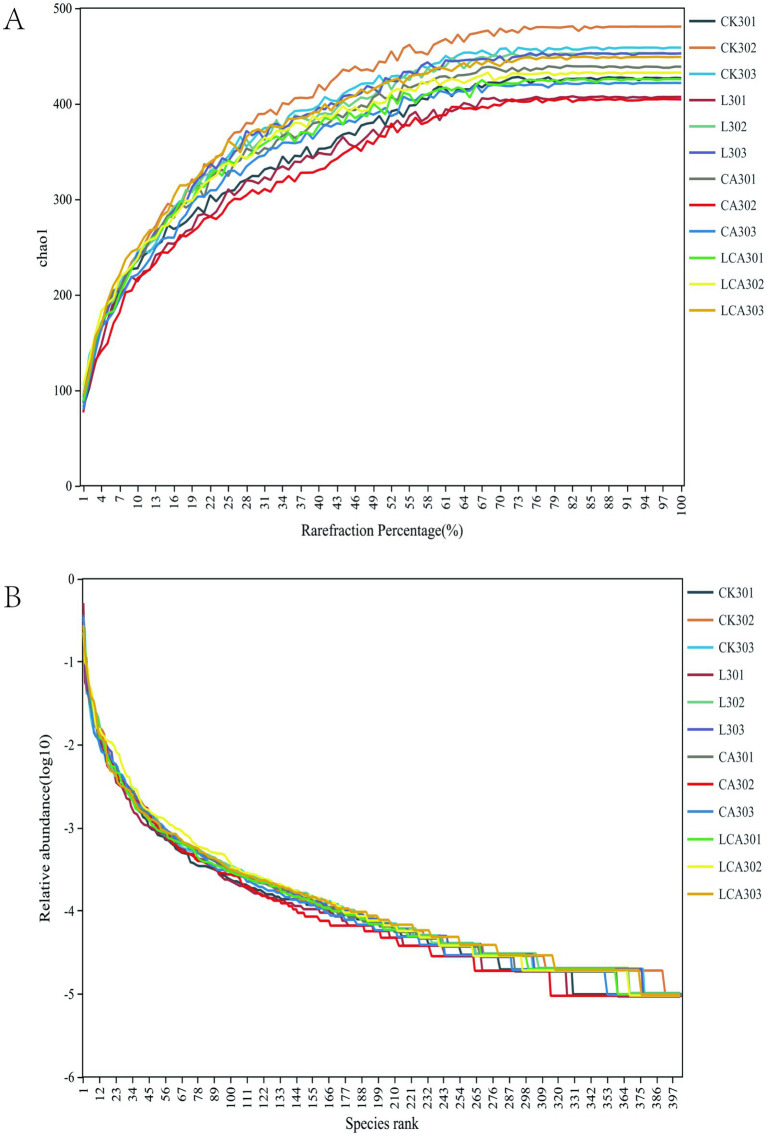
Dilution curves **(A)** and rank relative abundance curves **(B)** for different groups of king grass silage. Panel **(A)** shows dilution curves illustrating the relationship between sample dilution and observed diversity across the silage groups. Panel **(B)** presents rank relative abundance curves, displaying the distribution of species relative abundance, with the x-axis representing species rank and the y-axis their relative abundance. These curves compare microbial diversity and species richness among the silage groups.

Processing of the raw sequencing data yielded 1,231,788 valid sequences (an average of 102,649 per sample), representing a validity rate of over 95%. According to Figure 1A, the sequencing depth of all samples covered the majority of microorganisms, fulfilling the sequencing requirements. The rank abundance curve (Figure 1B) displayed the sample diversity, which meets the requirements for subsequent analysis.

**Figure 2:**
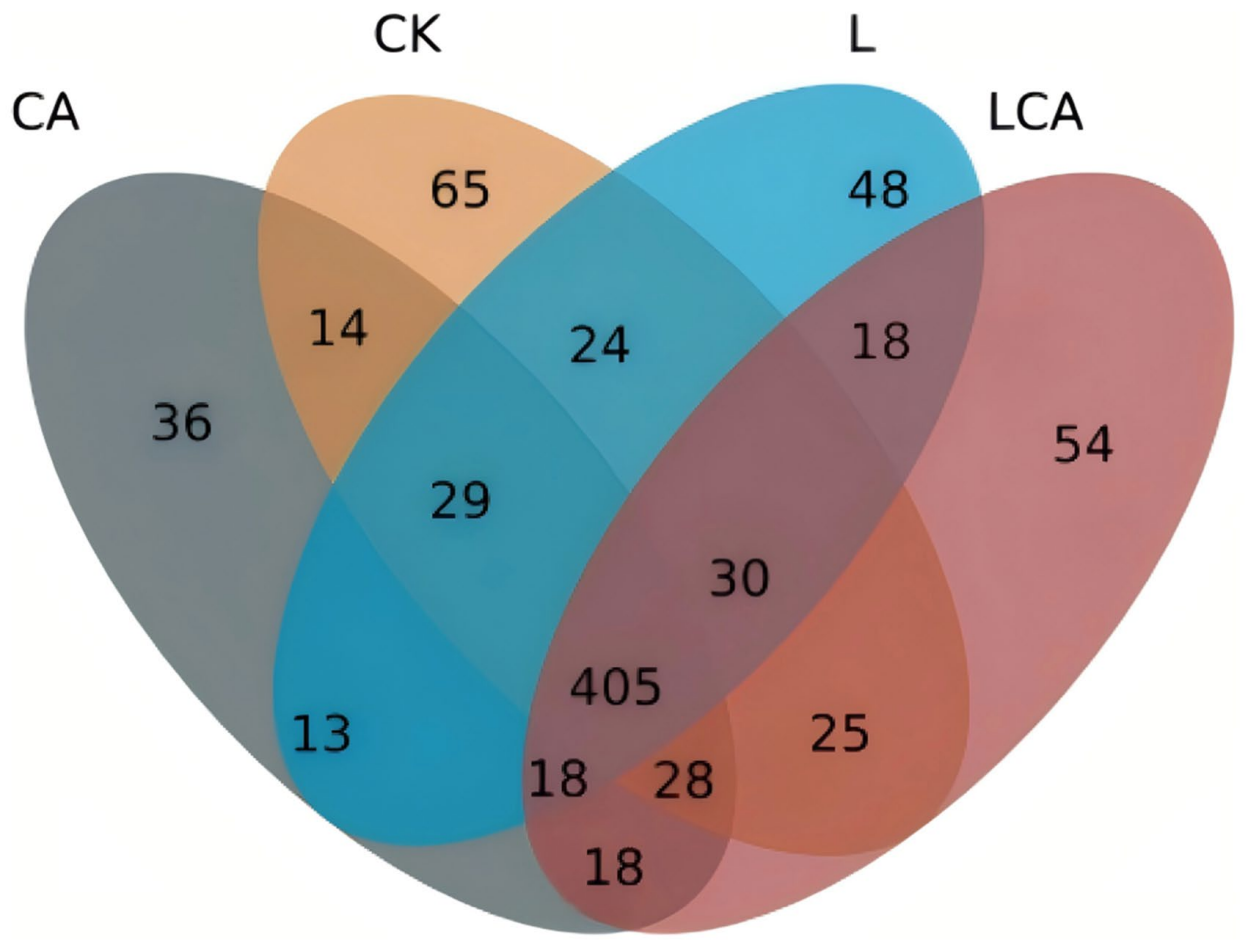
Venn diagram of OTUs (operational taxonomic units) shared between different treatment groups of king grass silage. The diagram highlights the overlap and unique OTUs among the treatment groups, showing the distribution of microbial taxa. The circles represent the distinct treatment groups, with the intersecting areas indicating common OTUs. This diagram provides a visual comparison of microbial community composition across the silage treatments.

The petal plots for different treatment groups of king grass silage were shown in Figure 2. The observed OTU counts were as follows: CK group, 620; L group, 549; CA group, 561; LCA group, 596. Among them, the CK and LCA groups had a higher number of OTUs. Additionally, Figure 2 showed that the number of core OTUs was 405.

#### 3.3.2 Alpha diversity analysis

The Alpha diversity indices for each sample were shown in Table 6. A high Shannon index indicated high species richness, while a low Simpson index suggested the presence of dominant species within a diverse microbial community. The Shannon index of the LCA group was significantly higher than that of the CK group (*p* < 0.05), while the Simpson index was significantly lower (*p* < 0.05). This indicated that the combined addition of *L. fermentum* and citric acid enhanced microbial richness and promoted dominant species in king grass silage. Furthermore, the Chao 1 and Ace indices in the CA group were significantly lower than those in the CK group (*p* < 0.05). However, there were no significant differences in the Chao 1 and Ace indices between the L, LCA, and CK groups (*p* > 0.05).

**Table 6:** Alpha diversity analysis of king grass silage.

Group	Chao1	ACE	Shannon	Simpson	Coverage
CK	455.37 ± 26.99^a^	528.67 ± 19.31^a^	2.77 ± 0.07^b^	0.18 ± 0.006^a^	0.99
L	437.5 ± 26.41^a^	501.24 ± 22.32^ab^	2.75 ± 0.27^ab^	0.19 ± 0.07^a^	0.99
CA	421.6 ± 17.1^a^	483.08 ± 15.34^b^	2.89 ± 0.06^ab^	0.14 ± 0.02^ab^	0.99
LCA	435.6 ± 11.93^a^	483.24 ± 15.83^ab^	3.11 ± 0.16^a^	0.11 ± 0.02^b^	0.99

Different lowercase letters indicate significant differences (*P* < 0.05) within the same column.

#### 3.3.3 Beta diversity analysis

**Figure 3:**
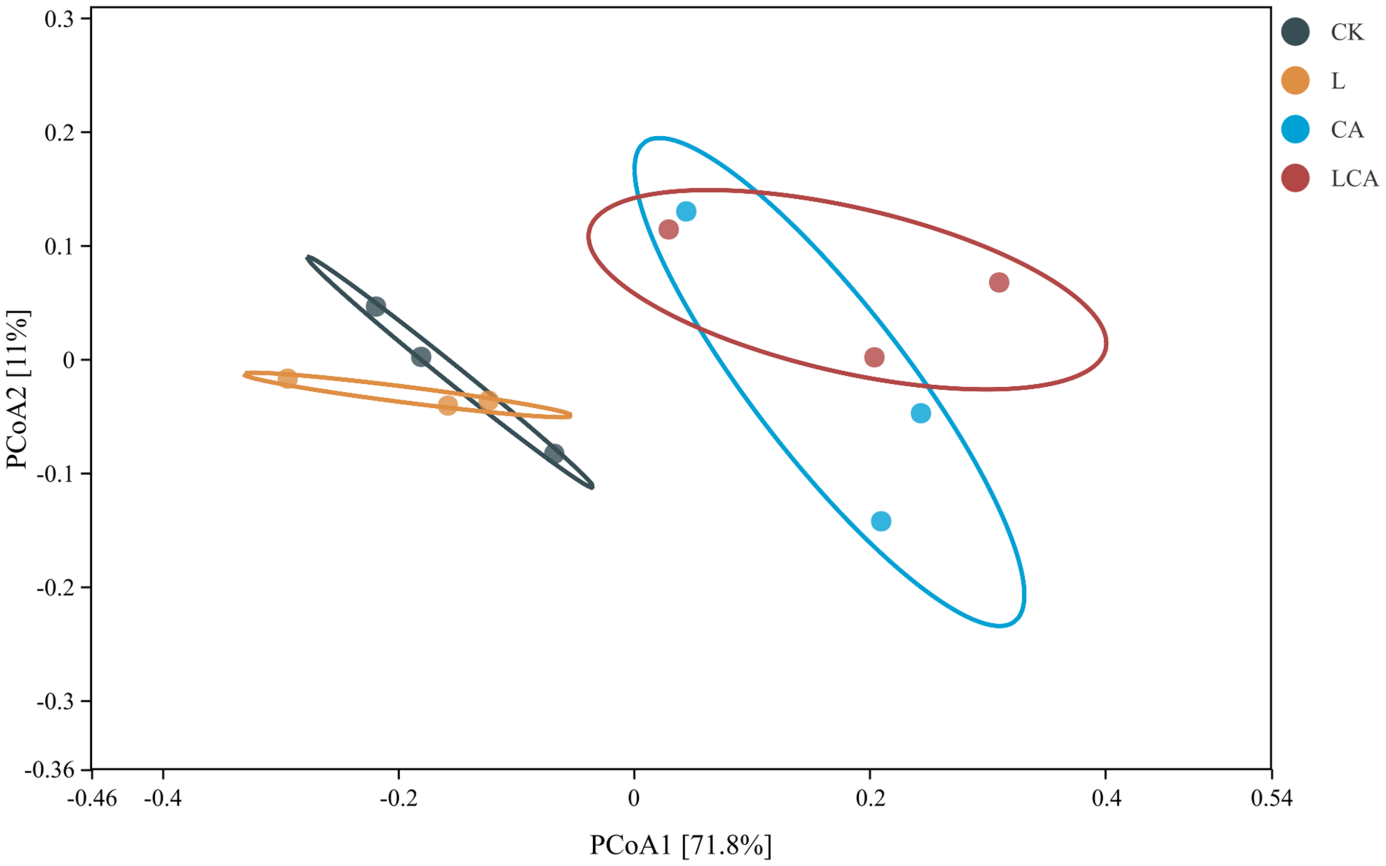
PCoA (Principal Coordinate Analysis) of king grass silage with different treatments. The plot visualizes the similarity and dissimilarity of microbial communities across treatment groups based on multivariate analysis of OTU composition. Each point represents a treatment group, with distances between points reflecting the degree of similarity in microbial profiles. The axes represent the principal coordinates that capture the most variation in the data, providing insights into the effects of different treatments on the microbial community structure.

Beta diversity is a method used to assess microbial community differences between samples based on the distance between species communities. Analyzing beta diversity revealed differences in the bacterial community composition of king grass silage. The principal coordinate analysis (PCoA) of differences of bacterial composition differences in king grass silage was shown in Figure 3. The first principal component (PCoA1) and the second principal component (PCoA2) accounted for 71.8 and 11% of the total variance, respectively. The bacterial compositions of the CK and L groups were located closely on the PCoA plot, indicating similarity in their bacterial compositions. The bacterial communities of the CK group were distinctly separated from those of the CA and LCA groups, indicating significant compositional differences, while CA and LCA exhibited highly similar bacterial profiles. Overall, the addition of *L. fermentum* alone did not significantly alter the bacterial composition of king grass silage, while citric acid or the combination of *L. fermentum* and citric acid significantly changed the composition.

#### 3.3.4 Analysis of bacterial community composition at the phylum level

**Figure 4:**
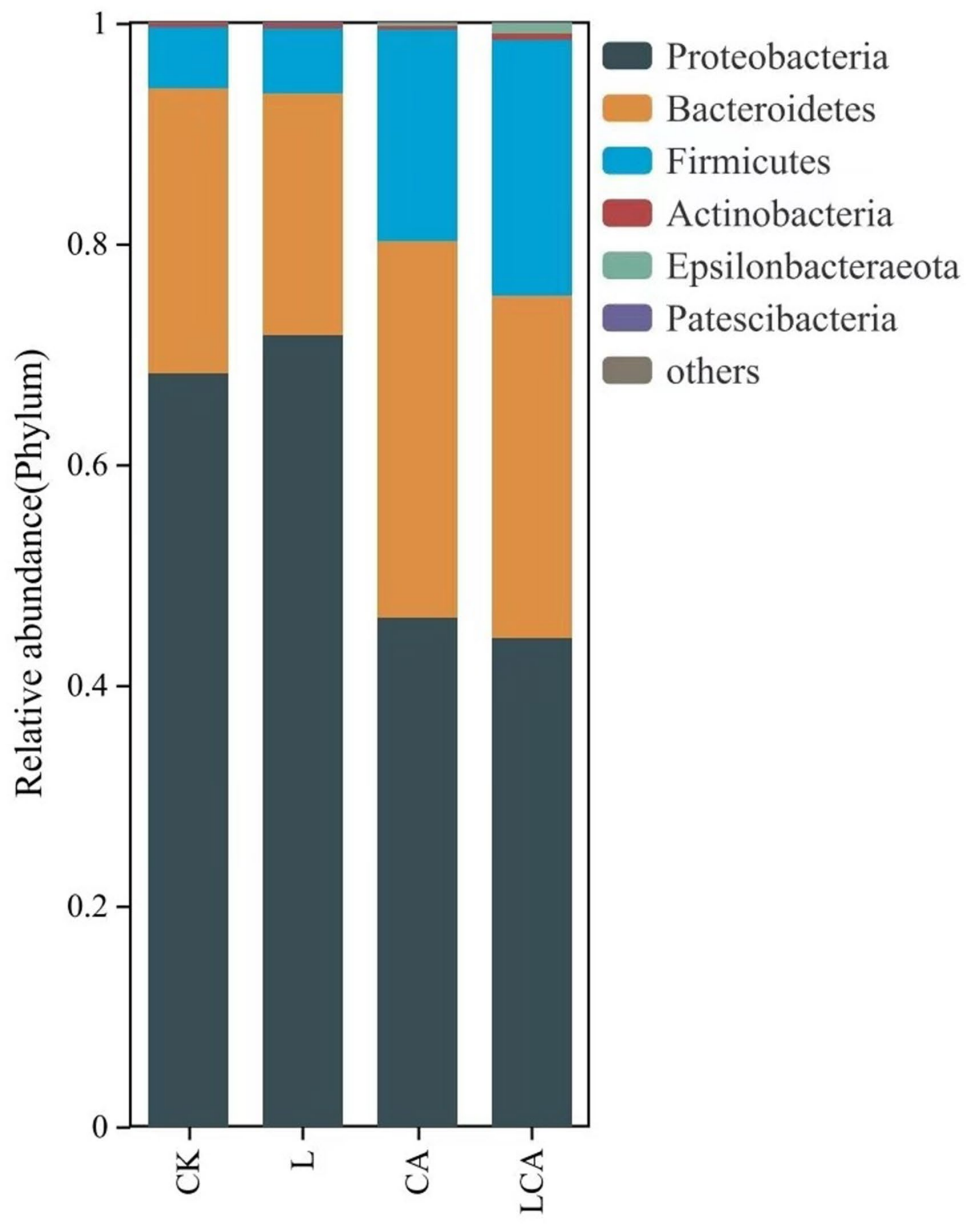
Relative abundance of species at the phylum level in different treatment groups of king grass silage. This bar chart illustrates the distribution of microbial phyla across the treatment groups, with the height of each bar representing the relative abundance of each phylum. The colors correspond to different phyla, providing a clear comparison of microbial composition between the groups. This analysis helps to reveal shifts in microbial community structure in response to different treatments.

The relative abundance of bacterial species at the phylum level in king grass silage under different treatments was shown in Figure 4. As shown in Figure 4, Proteobacteria, Bacteroidete, and Firmicutes were the dominant phyla in king grass silage. The relative abundance of Proteobacteria in the CK, L, CA, and LCA groups were 68.25, 71.74, 46.11, and 44.27%, respectively. The relative abundance of Bacteroidete were 25.79, 21.8, 34.01, and 31.06%, and those of Firmicutes were 5.59, 5.99, 19.25, and 23.1%. The addition of *L. fermentum* alone did not significantly alter the bacterial composition at the phylum level in wilted king grass silage. In contrast, the inclusion of citric acid, either alone or in combination with *L. fermentum*, notably modified the bacterial community structure, with the combined treatment exhibiting the most pronounced effect.

#### 3.3.5 Analysis of bacterial community composition at the genus level

**Figure 5:**
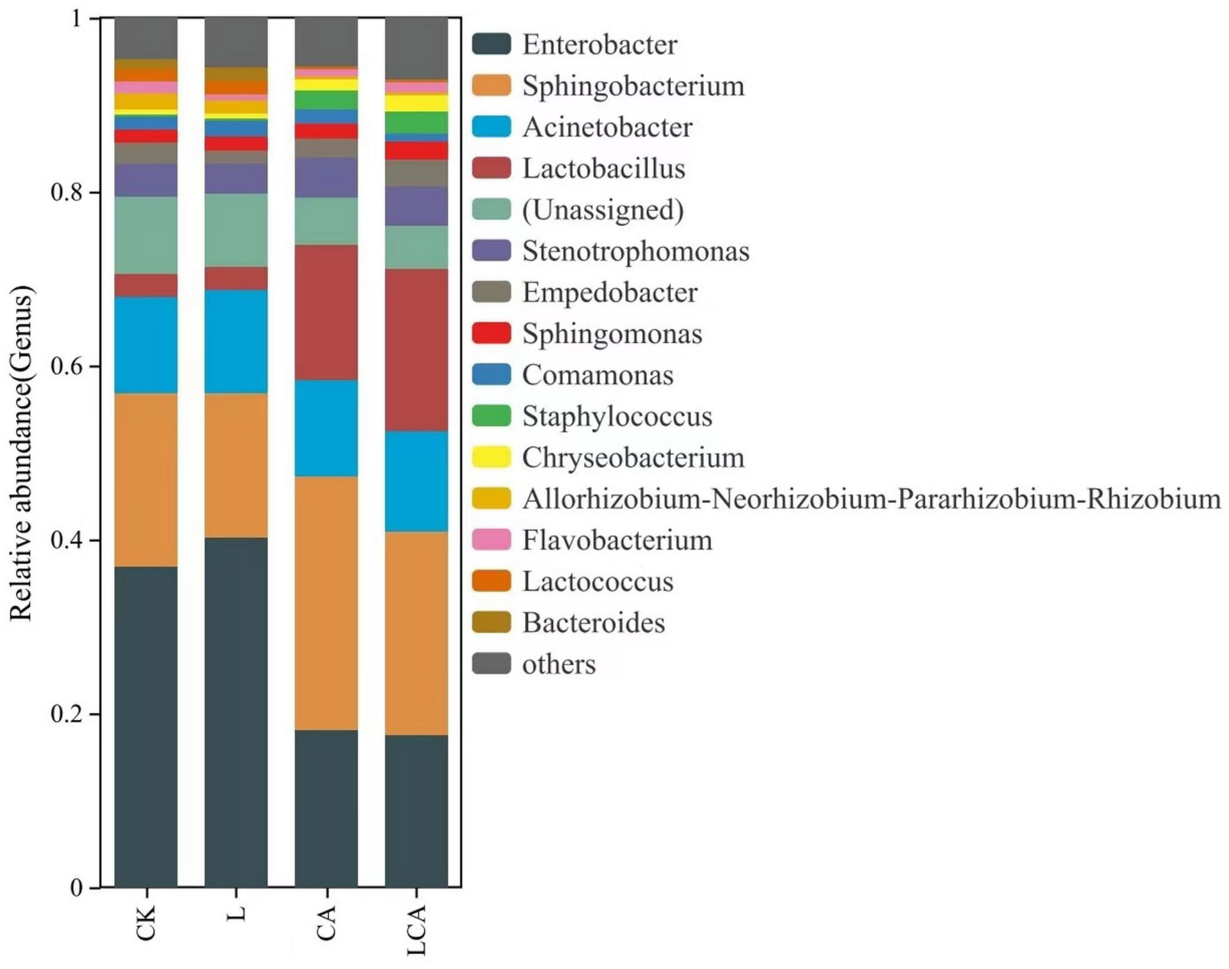
Relative abundance of species at the genus level in different treatment groups of king grass silage. The bar chart shows the distribution of microbial genera within each treatment group, with the length of each bar indicating the relative abundance of the corresponding genus. Different colors represent distinct genera, allowing for a clear comparison of microbial composition across the treatment groups. This analysis provides insights into the genus-level shifts in microbial communities due to the different treatments.

The relative abundance of bacterial species at the genus level in king grass silage under different treatments was shown in Figure 5. *Enterobacter*, *Sphingobacterium*, *Acinetobacter*, and *Lactobacillus* were the dominant genera in king grass silage across the different treatments. The relative abundance of *Enterobacter* in the CK, L, CA, and LCA groups were 36.92, 40.18, 18.13, and 17.44%, respectively. The relative abundance of *Sphingobacterium* in the CK, L, CA, and LCA groups were 19.85, 16.60, 29.09, and 23.51%, respectively. The relative abundance of *Acinetobacter* in the CK, L, CA, and LCA groups were 11.14, 11.96, 11.13, and 11.51%, respectively. The relative abundance of *Lactobacillus* in the CK, L, CA, and LCA groups were 2.66, 2.65, 15.53, and 18.65%, respectively. Compared to the CK group, the relative abundance of *Enterobacter* decreased in the CA and LCA groups, while that of *Lactobacillus* increased. In the L group, the relative abundance of *Enterobacter* increased, while *Lactobacillus* showed no significant change. This suggested that the addition of *L. fermentum* alone did not significantly increase the relative abundance of *Lactobacillus* in king grass silage, whereas the addition of citric acid alone or the combination of *L. fermentum* significantly increased its relative abundance. The main bacteria involved in silage fermentation were lactic acid bacteria. Comparative analysis revealed that the addition of lactic acid bacteria decreased the relative abundance of *Sphingobacteriu*m, while the addition of citric acid increased its relative abundance. Data comparison showed that the addition of *L. fermentum*, citric acid, or their combination in wilted king grass silage did not significantly alter the relative abundance of *Acinetobacter*. Additionally, *Lactococcus* was also detected in king grass silage.

### 3.4 Aerobic stability of king grass silage

**Figure 6:**
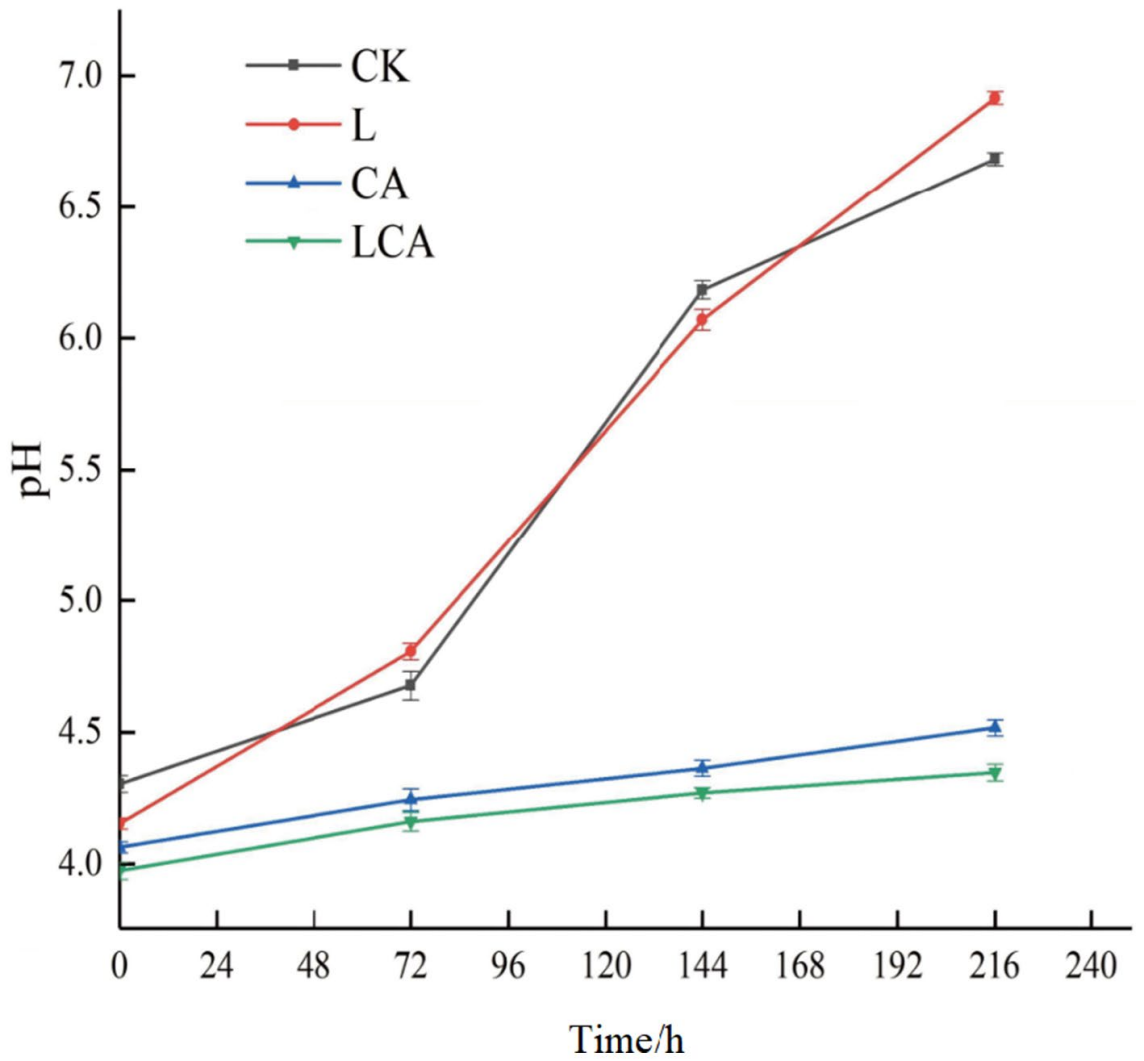
pH changes during aerobic exposure of king grass silage. This graph illustrates the variation in pH levels over time as the silage undergoes aerobic exposure. The x-axis represents the duration of exposure, while the y-axis shows the corresponding pH values.

Figure 6 illustrated the pH changes of king grass silage under different treatments during aerobic exposure. After the initiation of aerobic exposure, the pH values of all groups began to rise, starting at 72 h. The rate of pH increase was higher in the CK and L groups compared to the CA and LCA groups. After 216 h of aerobic exposure, the pH values for the CK, L, CA, and LCA groups were 6.68, 6.92, 4.51, and 4.35, respectively. Furthermore, the figure showed that the aerobic stability of the L group was significantly lower than that of the CK group (*p* < 0.05), while the aerobic stability of the LCA group was significantly higher than that of the CA group (*p* < 0.05). The analysis indicated that the addition of *L. fermentum* alone did not improve the aerobic stability of king grass silage, while the addition of citric acid or the combination of citric acid and *L. fermentum* significantly enhanced its aerobic stability.

## 4 Discussion

### 4.1 Effects of *Limosilactobacillus fermentum* and citric acid on the chemical composition of king grass silage

This study demonstrated that after wilting treatment, king grass silage exhibited a relatively high dry matter content. The moisture content of silage material was a critical factor affecting silage quality. Excessive moisture promoted the growth and proliferation of undesirable microorganisms (16). Research indicated that reducing the moisture content of silage material helped improve silage quality (17). In this study, the crude protein content in the CA and LCA groups was significantly higher than in the CK group, possibly because citric acid inhibited the hydrolysis of crude protein in king grass. Studies had shown that citric acid could inhibit the hydrolysis of crude protein in silage (13). This study further confirmed this hypothesis. The crude protein content in the L group did not differ significantly from the CK group, indicating that the addition of *L. fermentum* alone did not inhibit protein hydrolysis in king grass silage. These findings suggested that the combination of *L. fermentum* and citric acid could effectively improve protein preservation in silage. Excessive NDF content reduced feed intake in ruminants, while high ADF content affected digestibility. In this study, there was no significant difference in NDF content among the L, CA, LCA, and CK groups. However, the ADF content differed significantly, suggesting that *L. fermentum* and citric acid improved the digestibility of king grass silage for ruminants. WSC was the primary energy source for lactic acid bacteria fermentation during silage process. In this study, significant differences in carbohydrate content were observed among the L, CA, LCA, and CK groups. These findings suggest that *L. fermentum* and citric acid not only enhance the nutritional quality of the silage but also create a more favorable fermentation environment.

### 4.2 Effects of *Limosilactobacillus fermentum* and citric acid on the fermentation quality of king grass silage

The pH is an important indicator for assessing the fermentation quality of silage. The pH level reflected the quality of the silage. According to China’s silage standards, a pH of 3.4–4.4 indicated good silage quality (18). In this study, the pH of king grass silage was within this range, with the LCA group having a pH of 3.97. This suggested that the combined treatment of *L. fermentum* and citric acid contributed to a favorable fermentation environment. The lower pH of king grass silage could be attributed to three factors: (1) wilting reduced moisture content, inhibiting the growth of undesirable microorganisms and preventing nutritional competition with lactic acid bacteria; (2) the proliferation of lactic acid bacteria accelerated fermentation and increased lactic acid production, thereby inhibiting competing microorganisms; (3) citric acid had antimicrobial properties, and its lower pH further suppressed the growth of undesirable microorganisms.

Organic acids are another important indicator of silage quality. Lactic acid was the primary product of the silage fermentation process, and a higher lactic acid content generally indicated better quality silage (19). In this study, the lactic acid content in the CK, L, CA, and LCA groups differed significantly, with the LCA group showing the highest lactic acid content, suggesting that adding *L. fermentum* could increase lactic acid content, thereby improving silage quality. Additionally, studies have shown that appropriately adding citric acid could promote an increase in lactic acid content (20). This study further verified this viewpoint. The enhanced lactic acid production not only contributed to the overall quality of silage but also directly affected its preservation and digestibility. Lactic acid bacteria ferment water-soluble carbohydrates (WSC) to produce lactic acid, and the increase in lactic acid content resulted in a decrease in pH, which was also the main reason for the lower WSC content in the L, CA, and LCA groups. Acetic acid is the primary organic acid in tropical grass silage, and a higher concentration of acetic acid leads to an increase in pH (21). Moreover, research indicated that acetic acid content was positively correlated with the aerobic stability of silage (22). The significantly lower acetic acid content in the L group compared to the CK group is primarily attributed to the efficient fermentation driven by the added *L. fermentum.* This process rapidly consumed available WSC to produce lactic acid, lowering the pH and suppressing the activity of acetic acid-producing bacteria. Propionic acid and butyric acid are undesirable organic acids in silage, they damaged silage fermentation by stimulating Clostridium growth, resulting in nutrient loss and reduced palatability (23). Neither was detected in this study. Ammonia nitrogen is an important indicator for evaluating silage quality (24). The proteins in silage were hydrolyzed into ammonia nitrogen and other substances, thereby lowering the nutritional value and quality of the feed. In this study, the ammonia nitrogen content in the LCA group was significantly lower than that in the CK group, which was consistent with the crude protein content in the silage.

### 4.3 Effects of *Limosilactobacillus fermentum* and citric acid on bacterial composition in king grass silage

The silage process is a microbial fermentation process. High-throughput sequencing can reveal the microbial composition in silage and provide an in-depth analysis of the interactions between microbes during the silage process (25). In this study, the LCA group showed a lower Simpson index, indicating that the combined treatment with *L. fermentum* and citric acid increased bacterial diversity in king grass silage and promoted the dominance of specific beneficial taxa. *L. fermentum* and citric acid might have synergistically inhibited the growth of many undesirable microorganisms, with lactic acid bacteria becoming the dominant microbial group in the later stages of fermentation. Under anaerobic conditions, bacteria that could not adapt disappeared, resulting in increased bacterial diversity. Principal coordinate analysis (PCoA) results showed that the addition of *L. fermentum* alone did not significantly alter the bacterial composition of king grass silage, while the citric acid or *L. fermentum* + citric acid treatments significantly changed its bacterial composition. The PCoA results suggested that *L. fermentum* had a limited effect on microbial community structure. The similarity between CA and LCA groups implied that citric acid was the main factor influencing microbial composition.

In this study, the major bacterial phyla in king grass silage were Proteobacteria, Bacteroidete, and Firmicutes. After ensiling, the increase in Firmicutes and decrease in Proteobacteria could be ascribed to the anaerobic and acidic microenvironment in silage, which limited aerobic microorganisms and promoted LAB strains (26) The addition of *L. fermentum* alone did not significantly affect the major bacterial phyla in king grass silage, possibly due to the low moisture content after the wilting treatment, which inhibited the growth of lactic acid bacteria. This highlights the necessity of citric acid to drive significant changed in microbial composition. In the groups with citric acid or *L. fermentum* + citric acid treatment, the relative abundance of Firmicutes and Bacteroidete increased significantly. This result was consistent with previous studies (27). *Enterobacter* was considered a harmful bacterium in silage, capable of converting lactic acid into acetic acid and other substances (28). In this study, the relative abundance of *Enterobacter* was higher in the CK and L groups, but the acetic acid content was lower, possibly due to the reduced release of WSC from the king grass material with low moisture content, which inhibited its growth and metabolism. This study showed that the relative abundance of *Enterobacter* decreased after adding citric acid, which was consistent with other studies (12). Furthermore, after adding citric acid, the relative abundance of *Lactobacillus* increased, with higher relative abundance in the *L. fermentum* and citric acid combined group. The addition of citric acid seemed to have enhanced the growth of *Lactobacillus* while suppressing *Enterobacter*, a harmful bacterium. This phenomenon might be due to lactic acid bacteria using citric acid as a fermentation substrate. The acidic environment formed which was consistent with the growth of acid-sensitive microorganisms, thereby creating favorable conditions for lactic acid bacteria growth and forming a synergistic effect (29). *Sphingomonas* was a common genus in tropical grass silage (30), and researchers speculated it might be a characteristic genus of tropical silage (31). *Sphingomonas* was considered a beneficial bacterium in tropical grass silage due to its ability to degrade biogenic amines. *Acinetobacter* was an aerobic bacterium, but studies have shown that it can survive under anaerobic conditions containing acetic acid (32). Our previous studies also detected the presence of *Acinetobacter*. This study showed that the addition of *L. fermentum* and citric acid did not inhibit the growth of *Acinetobacter*.

### 4.4 Effects of *Limosilactobacillus fermentum* and citric acid on the aerobic stability of king grass silage

Aerobic stability is the ability of silage to resist spoilage from air exposure after fermentation is completed. Aerobic deterioration of silage feed can affect feeding effectiveness and, in severe cases, may damage the health of livestock and poultry. Aerobic deterioration is primarily caused by aerobic microorganisms, yeast, molds, and other microbes. Acetic acid can inhibit yeast growth, thereby alleviating aerobic spoilage of silage feed (33). In this study, the CA and LCA groups exhibited better aerobic stability, primarily due to their higher acetic acid content. After exposure to air, acetic acid inhibited the growth of some harmful microorganisms. The lactic acid bacteria added in this study were *L. fermentum*, which belongs to heterofermentative lactic acid bacteria. However, studies have shown that the addition of *L. fermentum* alone failed to improve the aerobic stability of silage. The possible reason for this phenomenon was that excessive wilting treatment resulted in excessively low moisture content, which not only inhibited the growth of undesirable microorganisms but also suppressed the growth and metabolism of *L. fermentum*.

## 5 Conclusion

In this study, *L. fermentum* HHL-5 was combined with citric acid as a silage additive and added to king grass silage. However, adding *L. fermentum* alone has a limited effect on improving the quality of wilted king grass silage, as low moisture content (<65%) inhibits the growth and metabolism of *L. fermentum*. Additionally, the results show that ensiling with *L. fermentum* combined with citric acid can significantly improve the quality of wilted king grass silage. Adding *L. fermentum* alone to wilted king grass has no significant effect on the microbial composition of king grass silage. However, ensiling with citric acid significantly alters the microbial composition, increased bacterial diversity, increasing the relative abundance of *Lactobacillus*, and decreasing the relative abundance of *Enterobacteriaceae*. In wilted king grass silage, adding *L. fermentum* alone does not improve aerobic stability, while ensiling with citric acid significantly enhances its aerobic stability.

## Data Availability

The sequencing data from this study have been submitted to the National Center for Biotechnology Information Sequence Read Archive database under Bioproject accession number of PRJNA1333438.
